# Editorial: Internet deviance

**DOI:** 10.3389/fpsyg.2024.1495767

**Published:** 2024-10-25

**Authors:** Elena Tsankova, Eric J. Vanman, Evita March

**Affiliations:** ^1^Department of Psychology, Institute for Population and Human Studies, Bulgarian Academy of Sciences, Sofia, Bulgaria; ^2^School of Psychology, University of Queensland, Brisbane, QLD, Australia; ^3^Institute of Health and Wellbeing, Federation University Australia, Berwick, VIC, Australia

**Keywords:** Internet, deviant behavior, trolling, cyberbullying, cybervictimization, problematic Internet use

## Introduction and objectives

Allowing for almost instantaneous audio-visual connection to others from anywhere around the globe, the Internet has become a favored communication medium in all major life domains—work, education, romantic relationships, etc. Certain features of the Internet, however, such as anonymity and untraceablity, afford opportunities for deviation from traditional ethical and moral norms. Cases of such Internet deviance include, yet are not limited to, trolling, cyberbullying, ghosting, stalking, and catfishing. The experience, both direct and vicarious, of these phenomena can have significant physical, psychological, and social impact.

Given this impact, Internet deviance is a highly relevant societal topic. The aim of this Research Topic was to gather and integrate recent findings in the field with the ultimate goal of identifying possible prevention and coping mechanisms. In particular, we wished to achieve better understanding within the following areas: motivations behind deviant Internet behaviors; evolution over time of deviant behavior on the Internet (for example, changes in netiquette, privacy policies, education as a result of instances of Internet deviance); cognitive and affective aspects of Internet deviance perception; standards associated with human and AI behavior online that may lead to perception of Internet deviance. The articles in the collection delve into many of these topics to varying degrees, but the central focus is placed on the motivation, causes, and consequences of several Internet behaviors that classify as “deviant”—trolling, cybervictimization-and-bullying, and problematic Internet use.

## Trolling

In an empirical study Nitschinsk et al. show that trolling on the Internet (intentionally seeking to deceive and aggress others; e.g., Buckels et al., 2019), both in terms of acceptance and perpetration, is not only driven by personality traits (sadism and psychopathy) but is also partially mediated by toxic motivations for anonymous self-expression online. By demonstrating that a property of the communication medium (i.e., anonymity) could benefit those engaging in trolling behavior. Nitschinsk et al. suggest that trolling may not be simply personality-driven as previously believed. The authors propose a functionalist perspective whereupon Internet anonymity may be a more likely predictor of online trolling than personality. This perspective not only advances the fundamental understanding of the trolling phenomenon, but also presents novel opportunities for the applied prevention of trolling by focusing, for example, on imposing limitations on online anonymity under certain conditions.

## Cybervictimization and cyberbullying

Drawing upon General Strain Theory (Agnew, 2006), Li and Peng demonstrate through binary logistic regression analyses that low self-control is associated with higher strain and increased likelihood of engaging in cyberbullying (purposeful act against an individual or a group on the Internet; e.g., Smith et al., 2008) for both cyberbullies cybervictims (those affected by cyberbullying; e.g., Betts, 2015). Importantly, regression and correlational analyses suggest that protective factors, such as constraints (i.e., factors that deter, inhibit, or dissuade criminal behavior; Agnew, 2005) and morality, may have mitigating effects on strain and engagement in cyberbullying behaviors. Insights for cyberbullying prevention stemming from this research are the design of strain-reduction-based and emotion regulation intervention methods such as, e.g., venting and reappraisal.

Further understanding on the cybervictimization-cyberbullying link is reached by Luo et al. who demonstrate through a chain mediation model that the predictive power of cybervictimization (becoming a victim of cyberbullying) for engagement in cyberbullying behavior is enhanced in a sequential fashion by stress and rumination. This newly uncovered mechanism suggests that optimal strategies for prevention of cyberbullying might include intervention at the post-victimization stress and rumination stages (e.g., by increasing family support). Thus, the model of Luo et al. presents opportunities to study cybervictimization and cyberbullying in terms of multi-factor systems that include the roles of social norms, groups, and institutions.

## Problematic Internet use

A meta-analysis by Zhu et al. systematically examines and integrates multiple findings (79 independent samples, 114,098 subjects) regarding problematic Internet use (with a specific focus on addiction) and the quality of parent-child relationship (relationship between primary caregivers and children; e.g., Schneider et al., 2017). Their findings show an overall negative association between the two variables. Further, this relationship was moderated by the type of Internet use (e.g., excessive use, general addiction, game addiction, mobile phone addiction, social media addiction, etc.), characteristics of parent-child relationship (e.g., communication, affinity), age, country, and alternative measures of problematic Internet use and parent-child relationship. These results pinpoint practical considerations for improving everyday parent-child relationships (e.g., ensuring better monitoring in countries with high parent-child power distance, enhancing social support at particularly vulnerable ages, etc.). Further, these findings provide fruitful directions for future study of the link between these relationships and problematic Internet use, such as differentiation between parenting roles, scale development, establishing links to personality, and more.

## Conclusions and outlook

The articles in this Research Topic provide insights of theoretical and applied nature about three central phenomena falling into the Internet deviance category—trolling, cybervictimization and cyberbullying, and problematic Internet use. These recent discoveries expand our understanding about deviant behavior on the Internet and outline directions for future work in the field. With recent technological advances in mind, we eagerly anticipate studies addressing human-AI interaction on the Internet and related perceptions of deviance. Amongst the most curious questions that arise in that respect would be one relating to perceived and applied “double standards”—would a human interaction partner be more willing to forgive or condemn AI engaging in behaviors that would be classified as “deviant” when attributed to another human?
